# Indicators of improved gestation housing of sows. Part I: Effects on behaviour, skin lesions, locomotion, and tear staining

**DOI:** 10.1017/awf.2023.47

**Published:** 2023-07-26

**Authors:** Martyna E Lagoda, Keelin O’Driscoll, Maria C Galli, Joanna Marchewka, Laura A Boyle

**Affiliations:** 1 Pig Development Department, Animal & Grassland Research & Innovation Centre, Teagasc Moorepark, Fermoy, Co Cork, Ireland; 2 Institute of Genetics and Animal Biotechnology of the Polish Academy of Sciences, Department of Animal Behaviour, ul. Postępu 36A, Jastrzębiec 05-552; 3 Department of Animal Medicine, Production and Health, University of Padova, Viale dell’Università 16, 35020, Legnaro (PD), Italy

**Keywords:** animal welfare, pig, rope, rubber, straw, chronic stress

## Abstract

Commercial gestation housing systems for sows generally fail to cater fully for their needs in terms of comfort or the ability to perform highly motivated behaviours, which can lead to chronic stress and an impairment to welfare. We compared a typical gestation system (CONTROL) with an IMPROVED one as regards oral stereotypies, aggressive behaviour, skin lesions, locomotion, and tear staining. Sows were mixed into 12 stable groups (six groups per treatment, 20 sows per group), 29 days post-service in pens with free-access, full-length individual feeding/lying stalls. CONTROL pens had fully slatted concrete floors, with two blocks of wood and two chains suspended in the group area. IMPROVED pens were the same but with rubber mats and a length of manila rope in each feeding stall, and straw provided in three racks in the group area. Direct observations of oral stereotypical (30 instantaneous scans per sow per day) and aggressive (all-occurrence sampling, 3 h per sow per day) behaviours were conducted 72 h post-mixing, in mid and late gestation. Skin lesions were counted 24 h and three weeks post-mixing, and in late gestation. Sows’ locomotion (locomotory ability) was scored using a visual analogue scale in mid and late gestation. Right and left eye tear staining was scored in late gestation. Indications of better welfare in IMPROVED sows included performance of fewer oral stereotypies in mid and late gestation, and lower tear stain scores. These sows performed more aggression in late gestation, which was associated with access to enrichment, but skin lesion counts were not affected. Thus, the changes made in the IMPROVED treatment benefitted aspects of sow welfare.

## Introduction

Sow welfare and productivity are negatively affected by the risk factors for chronic stress during gestation (Martinez-Miro *et al.* 2016; Lagoda *et al.* 2022). Many of these are associated with the physical environment, which can cause physical stress due to discomfort and pain, as well as psychological stress due to an inability to perform highly motivated species-specific behaviours (Jensen 1988; Lawrence *et al.* 1997; Stewart *et al.* 2008; Oczak *et al.* 2015). Building and pen designs are not easily altered, and changes are costly (Winkel *et al.* 2020). Nevertheless, sow welfare could be improved in conventional buildings through multiple smaller, incremental changes that may be more feasible for the pig producer and cumulatively may reduce chronic stress.

The widespread use of fully slatted concrete floors is a significant risk factor for sow welfare. These are uncomfortable to lie on (Tuyttens 2005; Spoolder *et al.* 2009), are associated with leg injuries and lameness (Elmore *et al.* 2010; Calderon Diaz *et al.* 2013), and preclude the provision of rooting material that could provide an outlet for investigatory behaviours (Tuyttens 2005). However, rubber floor mats can at least improve comfort while resting, and as a consequence reduce the risk of physical discomfort, thereby reducing stress (Boyle *et al.* 2000; Tuyttens *et al.* 2008; Elmore *et al.* 2010; Calderon Diaz *et al.* 2013; Ostovic *et al.* 2017). This is because the cushioning effect enables greater ease of changing posture, and reduces the risk of claw lesions and lameness (Boyle *et al.* 2000; Tuyttens *et al.* 2008; Elmore *et al.* 2010; Calderon Diaz *et al.* 2013).

Nevertheless, rubber mats do not provide the other benefits of straw bedding which is an outlet for many natural, species-specific sow behaviours, such as exploration, chewing, rooting and foraging (Tuyttens 2005; Stewart *et al.* 2008). Indeed, most conventional gestation housing systems inhibit expression of these highly motivated, natural behaviours in sows as a result of insufficient environmental enrichment (Calvert *et al.* 1996; van de Weerd & Ison 2019). Performance of such behaviours is important to the sow’s welfare (Studnitz *et al.* 2007). As well as providing an outlet for foraging behaviour, straw is also a high-fibre, ingestible material which can provide a degree of satiation to feed-restricted sows (Tuyttens 2005; Stewart *et al.* 2008). This can reduce stress and oral stereotypies resulting from unsatisfied feeding motivation (Whittaker *et al.* 1999; Edwards *et al.* 2019). Straw cannot be provided as bedding on slatted floors, but ‘off the floor’ structures such as racks and rooting towers are alternative delivery options (Stewart *et al.* 2008). While this limits the potential of straw as rooting material, it nevertheless remains an effective source of fibre and enrichment as it is investigable, manipulable, chewable/destructible, and edible (van de Weerd & Ison 2019), and also facilitates species-specific behaviours, such as exploration (Whittaker *et al.* 1999; Stewart *et al.* 2008).

Sows value fibrous enrichment resources such as straw very highly (Whittaker *et al.* 1999; Roy *et al.* 2019; van de Weerd & Ison 2019). Hence, a drawback of rooting towers and racks is the risk of competition (and thus sustained aggression), due to the potential for such structures to be monopolised by dominant individuals (Stewart *et al.* 2008). Nevertheless, this could be mitigated by strategic placement and provision of a number of straw delivery structures (Lagoda *et al.* 2021 [Au: 2021a], 2022). Natural fibre rope could also help satisfy sows’ behavioural motivation to chew, as it is destructible, chewable, manipulable, and investigable (Horback *et al.* 2016; Mkwanazi *et al.* 2019; van de Weerd & Ison 2019), with a potential positive effect on the performance of stereotypical behaviour (Casal-Plana *et al.* 2017). Indeed, Horback *et al.* (2016) showed that sows made contact with cotton rope more frequently than with rubber sticks or fixed woodblocks, and that this preference was observed day and night for two weeks. Providing rope in different locations to the straw dispensers could help to reduce competition around this valuable resource.

There are several methods of estimating chronic stress in sows (Lagoda *et al.* 2022), besides physiological measurements such as cortisol level (Herman *et al.* 2016; Carroll *et al.* 2018). One is through observation of stereotypical behaviours; these become established when animals are unable to cope with a challenge, or have no control over their environment (Martinez-Miro *et al.* 2016). For instance, oral stereotypies in sows are commonly used as an indicator of current or previous unsatisfied feeding motivation (Tatemoto *et al.* 2019). Tear staining (chromodacryorrhea) around the eyes is a rarely measured yet a promising method of estimating levels of chronic stress (DeBoer *et al.* 2015; Telkänranta *et al.* 2016; Larsen *et al.* 2019). The stain results from the secretion of porphyrin from the Harderian gland, and is thought to be under autonomic endocrine control of the hypothalamic-pituitary-adrenal axis and the sympathetic adrenomedullary system (DeBoer & Marchant-Forde 2013). Indeed, sows housed in free lactation pens had less tear staining around their left eye at weaning, than those confined to farrowing crates for the duration of lactation (Kinane *et al.* 2022).

There have been few studies investigating the combined effect of multiple minor adjustments to fully slatted pens on stress and welfare of pregnant sows (Elmore *et al.* 2011; Quesnel *et al.* 2019). However, this approach could provide additional benefits over research targeting individual risk factors for chronic stress (e.g. Stewart *et al.* 2008; Horback *et al.* 2016; Merlot *et al.* 2017), as in reality sows rarely experience stressors in isolation (Lagoda *et al.* 2022). Hence, the aim of this study was to investigate the effect of housing sows in a physically more comfortable and enriched environment on indicators of chronic stress and animal welfare. We hypothesised that sows housed in the improved environment would have lower levels of chronic stress reflected in reduced performance of oral stereotypies and lower levels of tear staining, culminating in better welfare compared to sows in the conventional pens.

## Materials and methods

### Ethical approval

Experimental work was authorised by the Teagasc Animal Ethics Committee (Approval no: TAEC 2020-266).

### Assignment of animals to trial, housing and management

This study took place on a 2,000-sow, commercial, farrow-to-finish farm in County Cork, Ireland, between July 2021 and April 2022. Oestrous was not synchronised on the farm. Sows (Large White × Landrace) were artificially inseminated in gestation stalls (2.30 × 0.55 m; length × width), at the onset of standing oestrous, and again within 24 h, and remained locked in stalls for 28 (28.9 [± 0.37]) days post-insemination. Sows went on trial over two three-week periods (period 1: 2–16 August 2021, where pens of replicate 1, 2, and 3 were formed; period 2: 15–29 November 2021, where pens of replicate 4, 5, and 6 were formed), whereby 40 served sows were enrolled in the study every week, with 20 sows assigned to conventional (CONTROL) and 20 sows assigned to treatment (IMPROVED) pens. In total, the study used 240 sows of parity 1 to 5 (mean [± SD]; 2.4 [± 1.03]) in six replicates. Sows were selected for the experiment on day 25 post-insemination out of approximately 60 to 80 sows per batch. Sows were restrained in gestation stalls to measure their back fat. Hair was shaved (and samples saved for hair cortisol analysis as part of a companion paper; Lagoda *et al.* 2023) from the dorso-lumbar region, identified by measuring 6.5 cm left and right from the mid-point at the spine marked by the position of the last rib. Back-fat depth (mm) was measured at the two identified sites using a Renco LEAN-MEATER® device [Renco; Minneapolis, MN, USA], and the average of the two values taken. The parity of each sow was noted, and overall health status evaluated, with those showing poor body condition and lameness excluded from the study. Blocks of two sows (i.e. 20 blocks per replicate) were created and balanced for back fat and parity and sows within each block were randomly assigned to either the CONTROL or IMPROVED treatment. The coefficient of variation for back fat was 25% in CONTROL, and 22% in IMPROVED, and for parity 42% in CONTROL, and 44% in IMPROVED.

**Figure 1.**
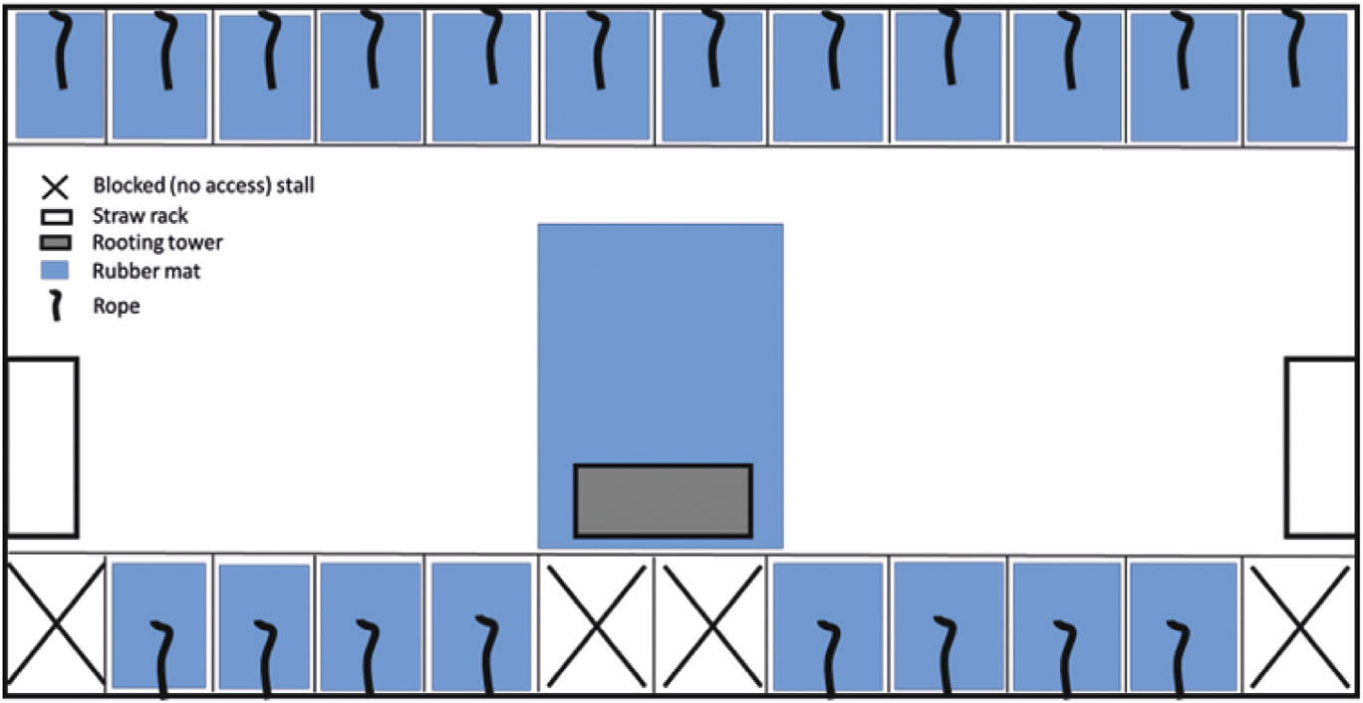
Diagram of the layout and set-up of the IMPROVED pen for pregnant sows.

The experiment started on the day that sows were moved to the gestation pens and mixed (day 28.9 [± 0.37] post-insemination) into their stable treatment groups. Each pen had 20 individual free access feeding/lying stalls (2.30 × 0.55 m; length × width), and sows were free to move around the remainder of the pen (7.3× 7.2 m; roaming area behind feeding stalls: 7.3 × 2.7 m). CONTROL pens had fully slatted concrete floors, two blocks of wood and two chains suspended within the group area. In replicates 4 to 6, pens also had a rubber toy (Astro 200, EasyFix Rubber Products, Ballinasloe, County Galway, Ireland) suspended from a chain. IMPROVED pens were the same, but with the addition of a length of natural fibre rope (1-m manila rope; Marine Suppliers & Co Ltd, Howth, Dublin, Ireland) suspended from the feed trough within each feeding stall, and straw provided from three custom-made structures (two straw racks at each end of the pen, and a rooting tower in the middle of the roaming area; Figure 1). Additionally, in the IMPROVED treatment, the slats in each feeding stall, as well as in front of the rooting tower, were covered with rubber mats (EasyFix Rubber Products, Ballinasloe, Co Galway, Ireland).

Sows were restrictedly fed (approximately 60–70% of *ad libitum* intake) a standard commercial, gestation liquid diet twice per day, and had *ad libitum* access to water via three nipple drinkers at one end of the pen.

### Behavioural observations

Sow behaviour was observed directly three times throughout pregnancy: 72 h post-mixing (day 33 [± 0.5]), in mid pregnancy (day 60 [± 4.1]), and in late pregnancy (day 106 [± 1.6]). There were three 1-h observation sessions per sampling day at the following times (scheduled to avoid feeding times and routine checks on the animals performed by farm staff): 0800–0900, 0930–1030 and 1230–1330h. Twenty-four hours prior to the observations, sows were identified by a number spray-marked onto their back to facilitate viewing from outside of the pen. The two observers practiced scoring behaviour until at least 90% intra- and inter-observer scores for repeatability were achieved prior to the onset of the study. Observers could not be blinded to the treatment. Pens of sows were scanned every 6 min (with the exception of replicate 1, 72 h post-mixing when scans were at 10-min intervals). Thus, in total, there were 30 recordings per sow per sampling day (with the exception of replicate 1; 72 h post-mixing there were 18 recordings per sow per sampling day). Observers switched between IMPROVED and CONTROL groups at the beginning of each session to balance the time spent observing each treatment. Behaviour, as well as posture (lying ventrally or laterally, standing, dog sitting) and location (group area, stalls) of each animal was recorded on every scan, based on an ethogram adapted from Cronin and Wiepkema (1984; Table 1).

**Table 1.** Ethogram for direct observations of behaviour of 240 sows by instantaneous scan sampling adapted from Cronin and Wiepkema (1984)

Detailed description of behaviour
Interaction with enrichment (chain/wood, Astro 200 toy, manila rope, straw rack, rooting tower, or straw on the ground)
Interaction with the floor (with little contact between rooting disc and floor, thus not defined as rooting; includes sniffing), pen barriers and stall railings, gates, but not with any enrichment item/device
Nosing, nibbling, sniffing, licking, chewing on another pig. Includes wound licking
Sniffing and exploring the observer from inside of the pen
Rooting (close contact between the rooting disc and the floor, and obvious pushing motion with the nose)
Standing still, with no other behavioural activity
Lying down, with no other behavioural activity. The animal’s eyes are still open
Sitting still, with no other behavioural activity
Sleeping (lying inactive with eyes closed)
Locomotion (walking, with no other behavioural activity)
Scratching against a wall or pen barrier
Chewing on a physical substance/object (pen fixtures, fittings, but not enrichment)
Eating (scored while the animal is inside the stall and has its head in the trough, with the sound of feeding/chewing)
Drinking (animal has the drinker in its mouth)
Urinating
Defaecating
*Oral stereotypies*
Sham chewing (in the absence of a physical substance/object)
Mouth stretching (opening of the mouth wider than when chewing/sham chewing)
Palate grinding (slight movement of the jaw, accompanied by a squeaky noise made by the movement of the tongue against the palate)
Sucking (movement of the mouth to create a circular shape with the lips, with an inward movement of air, sometimes accompanied by the sound of air being sucked in)
Tongue flicking (visible movements of the tongue outside of the mouth)
Licking of the floor, walls and pen barriers

### Stereotypical behaviour

On each scan, sows were observed for oral stereotypies, including: sham chewing, mouth stretching, palate grinding, sucking, tongue flicking, and licking. The proportion of scans (as a proxy for duration) sows spent performing oral stereotypies was calculated as a percentage of the total number of scans.

### Interaction with enrichment items

On each scan, sows were observed for their interaction with different types of enrichment (chain/wood, Astro 200 toy, manila rope, straw rack, rooting tower, straw on the ground). The proportion of scans (as a proxy for duration) sows spent interacting with enrichment items was calculated as a percentage of the total number of scans.

### Movement index

Data on the location of each sow in the pen (either in stalls or in the group area) on every scan was used to calculate a ‘movement index’, whereby a score of 1 was assigned each time a sow’s location changed between scans, with the sum of these scores representing a proxy for the amount of movement by a sow on each observation day.

### Aggressive behaviour

Aggressive behaviour of individual sows was recorded using all-occurrence sampling simultaneous to the observations of stereotypical behaviour, as well as sow posture and location in the pen, resulting in 3 h of observation per sow per sampling day (following the same scoring arrangement as for behavioural observations, with observers switching between IMPROVED and CONTROL groups at the beginning of each session to balance the time spent observing each treatment). An ethogram of the aggressive behaviours recorded was adapted from Stewart *et al.* (2008; Table 2). The pen location (stalls, group area) in which aggressive behaviours took place was also recorded. Occurrences of all aggressive behaviours were summed to yield a total count of aggressive behaviours per sow for each sampling day. Aggressive behaviours were also categorised as either ‘non-contact’ (chasing, threat, avoidance), or ‘contact’ (fighting, biting, head knocking, chasing with vulva biting), and summed to yield a total count per sow for each category on each sampling day.

**Table 2.** Ethogram of aggressive behaviours adapted from Stewart et al. (2008)

Behaviour	Detailed description
Aggressive biting^1^	Biting any part of another sow (except vulva), but not as a part of head thrust
Vulva/anogenital region biting	Biting the vulva/anogenital region of another sow whilst chasing her
Head knocking^1^	Ramming or pushing another sow with the head (with or without biting)
Fighting^1^	Mutual pushing parallel or perpendicular, ramming or pushing of the opponent with the head, with or without biting in rapid succession. Lifting the opponent by pushing the snout under its body
Chasing^2^	Moving rapidly in pursuit of another sow
Threat^2^	Being in head-to-head contact with another sow
Avoidance^2^	Sow actively withdrawing in response to head-to-head threat of another sow

1 ‘Contact’ aggressive behaviour

2 ‘Non-contact’ aggressive behaviour

### Skin lesions

Skin lesions were counted 24 h post-mixing, three weeks post-mixing (day 52 [± 0.4]) and in late pregnancy (day 109), following a method validated by Turner *et al.* (2006). Skin lesions were counted on the anterior (head, neck, shoulders, front legs), middle (flanks, back), and posterior (rump, hind legs), on the left and right sides of the body. Counts included fresh skin lesions only, identified by colour and the estimated age of scabbing. The length or diameter of skin lesions was not weighted. All counts were summed to calculate a total skin lesion count for each sow per inspection.

### Locomotion

To score locomotion (locomotory ability), sows were encouraged to take at least six strides on the fully slatted, concrete floors of the group pen in mid (day 57.3 [± 0.82]) and in late (day 108) pregnancy, using a visual analogue scale (VAS) developed by Lagoda *et al.* (2021b). The scale consisted of a 150-mm horizontal line. Locomotion was scored by marking a point along the scale, with increasing impairment represented by a mark further to the right of the line (0 mm representing perfect locomotion, and the very right end, 150 mm representing severely impaired locomotion). The distance from the left-hand end of the scale was measured and the value for each recorded in millimetres. Thus, the greater the number, the more impaired the locomotion.

### Tear staining

Tear staining was scored for each sow in late pregnancy (day 103 [± 0.5]), on the right and left eye separately, according to a scale developed by DeBoer *et al.* (2015; Table 3).

**Table 3.** Sow tear-stain scoring system (DeBoer et al. 2015)

Description of tear stain	Score
No visible stains	0
Barely detectable stains, not extending below eyelid	1
Visible stain, < 50% of the size of the eye	2
Visible stain, 50–100% of the size of the eye	3
Visible stain, > 100% of size of the eye, but not extending below the mouth line	4
Visible stain, extending below the mouth line	5

### Statistical analysis

SAS v9.4 was used for all statistical analyses (SAS Inst Inc, Cary, NC, USA) with sow or pen as the experimental unit depending on the analysis. Differences were reported when *P* ≤ 0.05, while statistical trends were reported when *P* > 0.05 and *P* ≤ 0.10. Residuals were checked for normality by examination of histograms, quantile-quantile and normal distribution plots using the univariate procedure. Degrees of freedom were estimated using the Kenwood-Rogers adjustment, and *P*-values adjusted using the Tukey-Kramer adjustment where mixed models were used. Data are presented as least square (LS) means and standard errors (SE).

All general linear models included the interactive effect of treatment and time, as well as replicate, time as a repeated effect, and pen as a random effect. Covariance structure was selected on the basis of best fit, using the minimum finite-sample corrected Akaike Information Criteria (AIC). Further details of each model are described below.

Oral stereotypy % (proportion of scans sows were performing stereotypies as a percentage of the total number of scans), skin lesion counts, locomotion scores, and movement index were analysed using general linear models (PROC MIXED) and individual sow data used in the analysis (sow as experimental unit).

Enrichment interaction % (proportion of scans sows interacted with different enrichment items as a percentage of the total number of scans) was analysed using general linear models (PROC MIXED) which included the interactive effect of enrichment type, treatment and time, and the repeated effect of time. Sow was used as the experimental unit.

The sum of all aggressive behaviours for each sow were averaged per pen (pen as experimental unit) to normalise the data which were also analysed using general linear models (PROC MIXED). We conducted two analyses. The first investigated the interaction between treatment, time and location in the pen, and the second the interaction between treatment, time and aggression type.

The proportion of scans sows spent in different pen locations and postures was used as a proxy for time spent in different pen locations and postures. Here, individual sow values were averaged per pen (pen as experimental unit) and summed for each location and posture to normalise the distribution of the data. Location and posture were then analysed separately using general linear models (PROC MIXED). The first model included the interaction between treatment nested within location, and time nested within location, and the second the interactive effect between treatment nested within posture, and time nested within posture.

The Mann-Whitney test (PROC Npar1Way) was used to compare tear stains for both the right and left eyes of sows from CONTROL and IMPROVED pens, in late pregnancy. Sow was used as the experimental unit. Right and left eyes were analysed separately, as previous work showed differences in tear staining for both eyes in response to stressors (DeBoer *et al.* 2015).

## Results

### Oral stereotypies

There was an interaction between treatment and time on performance of oral stereotypies (*P* = 0.001; Table 4). There was no effect of treatment at 72 h post-mixing (*P* > 0.05; Table 4). However, sows in CONTROL pens performed more oral stereotypies than sows in IMPROVED pens in mid (*P* < 0.001) and late (*P* < 0.001; Table 4) pregnancy.

**Table 4.** Differences (least square means [± SEM) over time in oral stereotypies, total aggressive behaviour, skin lesion counts, locomotion and the movement index of 240 sows housed in either conventional (CONTROL; n = 120) or treatment (IMPROVED; n = 120) pens

Variable	CONTROL	IMPROVED	*P*-value	Overall *P*-value (Treatment × Time)
Oral stereotypy (Proportion of scans, % of total number of scans)				
72 h post-mixing	7.91 (± 1.12)	5.87 (± 1.12)	0.790	0.001
Mid pregnancy	14.27 (± 1.12)	6.27 (± 1.12)	< 0.001	
Late pregnancy	12.19 (± 1.12)	4.94 (± 1.13)	< 0.001	
Total aggression (Sum of counts of sow aggressive behaviours averaged per pen)				
72 h post-mixing	0.55 (± 0.11)	0.44 (± 0.11)	0.984	0.018
Mid pregnancy	0.40 (± 0.11)	0.48 (± 0.11)	0.997	
Late pregnancy	0.42 (± 0.11)	0.96 (± 0.11)	0.016	
Total skin lesion count				
24 h post-mixing	21.88 (± 1.10)	20.46 (± 1.10)	0.942	0.933
3 weeks post-mixing	10.65 (± 1.10)	9.19 (± 1.10)	0.936	
Late pregnancy	7.95 (± 1.10)	7.22 (± 1.11)	0.997	
Locomotion (mm)				
Mid pregnancy	15.8 (± 1.1)	13.8 (± 1.1)	0.781	0.946
Late pregnancy	15.5 (± 1.1)	14.1 (± 1.1)	0.946	
Movement index (No of location changes)				
72 h post-mixing	2.63 (± 0.22)	2.62 (± 0.22)	1.000	0.003
Mid pregnancy	3.58 (± 0.22)	4.76 (± 0.22)	0.003	
Late pregnancy	3.03 (± 0.22)	4.08 (± 0.22)	0.013	

### Interaction with enrichment items

**Figure 2.**
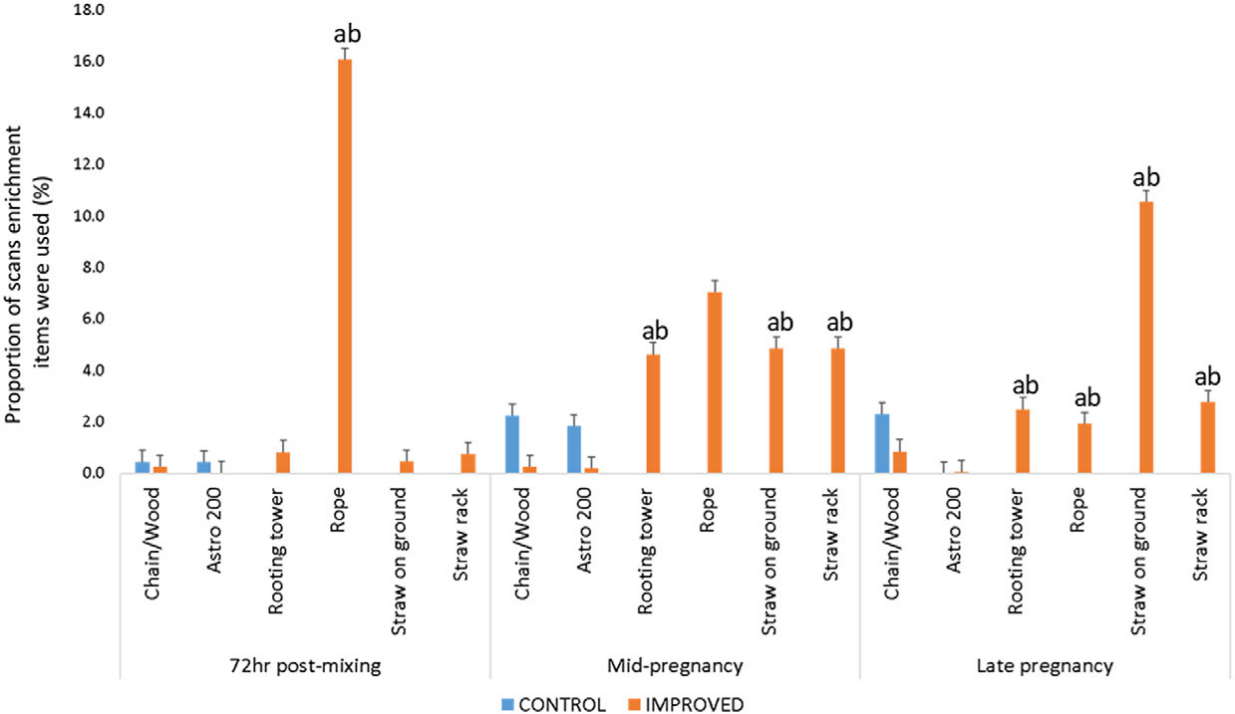
Proportion of scans (% of total number of scans, as proxy for duration) sows in IMPROVED and CONTROL groups spent interacting with different enrichment items during behavior observations 72hr post-mixing, and in mid and late pregnancy. ^a, b^ Significant differences between treatments within time, and enrichment items. Error bars represent standard error.

There was an interaction between treatment, time and enrichment item on the % of scans sows spent (proxy for duration) interacting with enrichment items (*P* < 0.001; Figure 2). There were no differences in the level of use of chain/wood and Astro 200 toy between treatments at any time-point. Understandably, straw (rack, rooting tower, straw on the ground) and manila rope were the most often used enrichment items in the IMPROVED pens (*P* < 0.05; Figure 2). While IMPROVED sows began and continued to interact with rope from 72 h post-mixing (immediately after mixing), meaningful interactions with straw enrichment were only recorded from mid pregnancy onwards (*P* < 0.001 for each enrichment item; Figure 2).

### Location and posture of sows during behaviour observations

**Figure 3.**
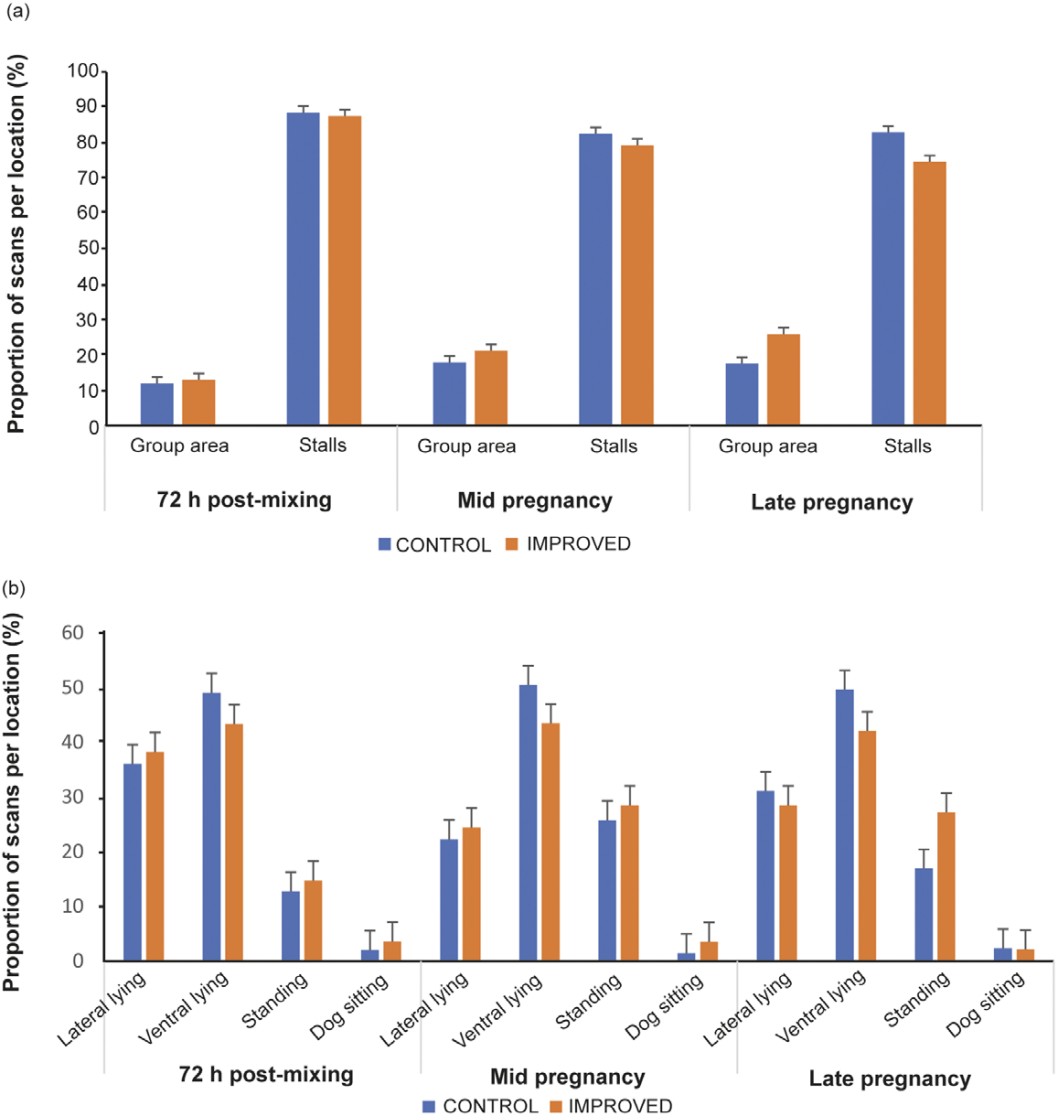
Proportion of scans (% of total number of scans, as proxy for duration) spent in different (a) pen locations and (b) postures by sows in IMPROVED and CONTROL pens during behavioural observations 72 h post-mixing, and in mid and late pregnancy. No significant differences between treatments within time, and location or posture were recorded. Error bars represent standard error.

There tended to be an interaction between treatment, time and location of sows during behavioural observations (*P* = 0.086; Figure 3). In late pregnancy, sows in the IMPROVED pens tended to spend more time in the group area (*P* = 0.081), and tended to spend less time in the stalls (*P* = 0.081) compared to sows in CONTROL pens (Figure 3). However, sows in both treatments spent more time inside the stalls than in the group area as pregnancy progressed (*P* < 0.05; Figure 3).

No interaction was found between treatment, time and the posture of sows (*P* = 0.952; Figure 3). However, there was an effect of time on sow posture, with sows spending most time lying ventrally throughout pregnancy (*P* < 0.001; Figure 3).

### Movement index

There was an interaction between treatment and time on the movement index (*P* = 0.003; Table 4). Although there was no effect of treatment 72 h post-mixing (*P* > 0.05), in mid and late pregnancy, sows in the IMPROVED pens had a higher movement index than CONTROL sows (Mid: *P* = 0.003; Late: *P* = 0.013; Table 4).

### Aggressive behaviour

There was also an interaction between treatment and time when it came to total aggressive behaviours (*P* = 0.018; Table 4). Although there was no difference 72 h post-mixing and in mid pregnancy (*P* > 0.05), there were more aggressive behaviours among sows in the IMPROVED pens than in CONTROL pens in late pregnancy (*P* = 0.016; Table 4).

### Location of aggressive behaviour

**Figure 4.**
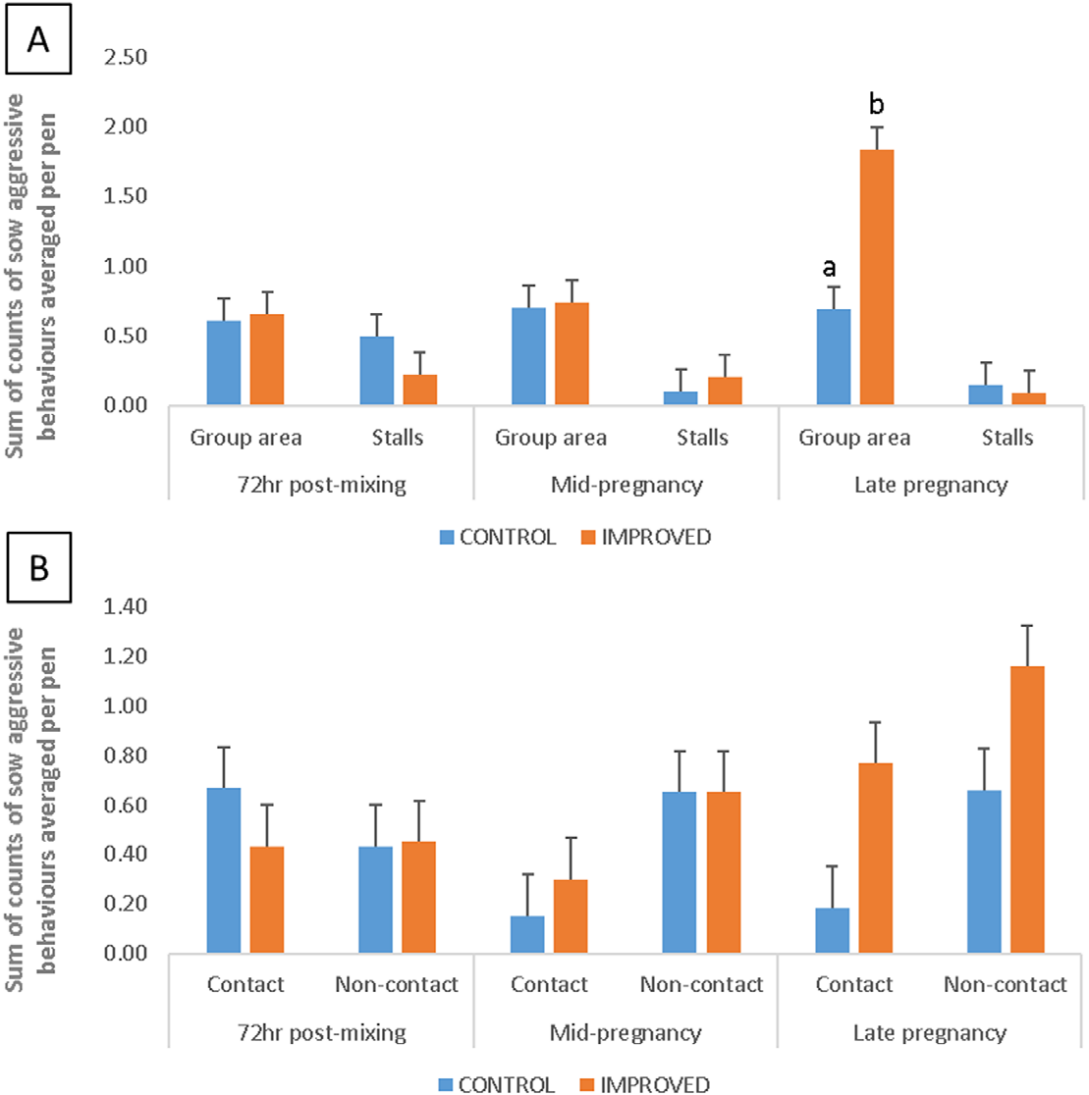
Differences (Least square means ± standard error; SE) in aggressive behaviour A) levels in group and stall area, and B) type (non-contact or contact), among sows in IMPROVED (n = 120) and CONTROL (n = 120) pens 72hr post-mixing, and in mid and late pregnancy. ^a, b^ Significant differences between treatments within time, and location or type of aggression. Error bars represent standard error.

There was an interaction between treatment, time and the location of aggression in the pen (*P* = 0.023; Figure 4). Levels of aggression were higher in the group area of IMPROVED pens compared to CONTROL pens in late pregnancy (*P* < 0.001; Figure 4). In addition, there were higher levels of aggression in the group area compared to the stalls of the IMPROVED pens in late pregnancy (*P* < 0.001), whereas this difference was not significant in CONTROL pens.

### Type of aggressive behaviour

Overall, there was no interaction between treatment, time and type of aggression (*P* = 0.672; Figure 4). However, there was an effect of type of aggression, with sows experiencing more non-contact than contact aggression throughout pregnancy in both IMPROVED and CONTROL pens (*P* = 0.014; Figure 4).

### Skin lesion counts

Generally, skin lesion counts were low throughout pregnancy (IMPROVED 12.3 [± 12.66]; CONTROL 13.5 [± 14.21]). There was no interaction between treatment and time, and no effect of treatment on total skin lesion counts at any point throughout pregnancy (*P* > 0.05; Table 4).

### Locomotion

Sows were considered lame if they scored 60 mm or higher (≥ 60) on the VAS. Locomotion scores were low throughout pregnancy (IMPROVED 7.0 [± 10.65]; CONTROL 7.9 [± 12.81]), with low occurrence of lameness (CONTROL n = 4; IMPROVED n = 2 lame sows). There was no interaction between treatment and time, or effect of treatment on locomotion at any point throughout pregnancy (*P* > 0.05; Table 4).

### Tear stain score

Sows in the IMPROVED pens had lower tear stain scores for both right and left eyes in late pregnancy compared to sows in CONTROL pens (*P* < 0.001 for each; Tables 5 and 6).

**Table 5.** Wilcoxon scores (rank sums) for tear stain scores on the right and left eyes of sows in IMPROVED and CONTROL pens in late pregnancy, calculated using the Mann-Whitney *U* test

	Sum of scores	Expected under H0	Standard deviation	Mean score	*U*-statistic	*P*-value
Right eye						
IMPROVED	11,834	14,101	508.7	100.3	11,834.0	< 0.001
CONTROL	16,607	14,340	508.7	138.4		
Left eye						
IMPROVED	12,087.5	14,101	510.8	102.4	12,087.5	< 0.001
CONTROL	16,353.5	14,340	510.8	136.3

**Table 6.** Number of sows per tear-stain score category around right and left eye in late pregnancy

		CONTROL			IMPROVED		
	Tear stain score	N of sows per treatment	N sows per score	% of all sows	N of sows per treatment	N sows per score	% of all sows
Right eye	0	120	4	3	120	11	9
	1	120	24	20	120	45	38
	2	120	46	38	120	40	33
	3	120	32	27	120	20	17
	4	120	12	10	120	1	1
	5	120	2	2	120	1	1
Left eye	0	120	5	4	120	13	11
	1	120	28	23	120	39	33
	2	120	38	32	120	44	37
	3	120	34	28	120	19	16
	4	120	12	10	120	3	3
	5	120	3	3	120	0	0

## Discussion

Current gestation housing systems pose multiple risks for chronic stress in sows (Salak-Johnson 2017; Lagoda *et al.* 2022), and it is difficult to eliminate/ameliorate them all (Winkel *et al.* 2020) within the constraints of existing commercial buildings. We implemented simultaneous minor modifications to conventional fully slatted gestation pens with the aim of improving sow welfare. The results of the current study provide good evidence that even the minor modifications that were made to a typical conventional system design in the IMPROVED treatment enhanced the physical and psychological comfort of pregnant sows. The positive effects on oral stereotypies, tear stains, and also the lack of negative impacts on locomotion despite increased levels of movement, suggest that the incremental improvements made in the pens did have an additive positive effect on overall levels of sow welfare.

As predicted, both forms of enrichment were the target of sows’ investigatory behaviour. Sows in IMPROVED pens not only investigated, but also ingested the straw (ME Lagoda, personal observation 2022, as based on the rate of disappearance of straw, as well as lack of evidence of it beneath the racks, or floating in the slurry beneath the slats), which likely provided additional gut fill, and increased satiety (Stewart *et al.* 2008). In line with Whittaker *et al.* (1998) and Bernardino *et al.* (2021), this was reflected in substantially lower levels of oral stereotypies both in mid and late pregnancy compared to CONTROL sows. Although inedible and perhaps not the ideal form of enrichment for sows, the manila rope in the individual stalls likely also contributed to this effect (Casal-Plana *et al.* 2017) by providing an outlet for oral stereotypies or by redirecting their sham-chewing behaviour. This was reflected in sows’ high level of interaction with the ropes in the individual stalls. Hence, the additive effects of straw and rope provision in IMPROVED pens was likely beneficial to the sows’ psychological and physiological needs, and thereby reduced levels of chronic stress.

There is a possibility that the tendency for increased time out of stalls in the IMPROVED sows could indicate a lower preference for the rubber mats provided inside the stalls. However, placing this result in the context of the presence of enrichment in the group area, it is more likely that sows were encouraged out of the stalls to interact with enrichment sources which they valued highly, reflected in high levels of interaction with the straw resources. It is also in line with the levels of aggression recorded in the IMPROVED pens.

Levels of aggression recorded in late pregnancy were higher in the IMPROVED pens, and particularly in the group area compared to the stalls. Based on the high levels of sow interaction with enrichment items provided in the group area of the pen, this likely reflects competition between the sows for access to those sources of enrichment material (Stewart *et al.* 2008). Contrary to our expectations, providing three spatially separated points of straw delivery did not ameliorate competition for access to these resources.

Aggressive behaviours performed by sows in the IMPROVED and CONTROL pens were mostly threat and avoidance based (i.e. there was little physical contact between the sows). Therefore, it is not surprising that in spite of the higher levels of aggression recorded in late pregnancy there was no treatment effect on skin lesion counts at any stage. This is in line with Stewart *et al.* (2008) and Horback *et al.* (2016) and suggests that even with fewer sources of enrichment the aggression associated with competition for access to such resources is less severe compared to aggression associated with competition for access to food in feed-restricted sows (Horback *et al.* 2016).

Sows in the IMPROVED pens moved around the pen more than CONTROL sows in mid and late pregnancy. Arguably, their higher movement index could have been detrimental for hoof health given that the majority of the pen flooring consisted of concrete slats which is a major risk factor for lameness, especially when combined with higher levels of agonistic behaviour (Philipot *et al.* 1994; Calderon Diaz & Boyle 2014). However, there was no negative effect on the locomotion of sows in the IMPROVED pens. Indeed, more exercise (Perrin & Bowland 1977; Marchant & Broom 1996; Schenck *et al.* 2008) combined with better comfort while lying provided by the rubber mats (Boyle *et al.* 2000; Elmore *et al.* 2010; Calderon Diaz *et al.* 2013) should possibly have improved the locomotory ability of sows in the IMPROVED pens. The absence of a difference between the treatments probably reflects the low levels of lameness in general for this sow herd. Moreover, sows in both treatments spent more time inside the stalls than in the group area throughout pregnancy, which is typical for this housing system (Olsson *et al.* 1993; Arey & Edwards 1998). Indeed, in the IMPROVED pens, this meant that sows spent a greater proportion of scans, and therefore more time on the rubber flooring which could have compensated for any potential detrimental effect of the increased movement around the slatted concrete of the group area.

Lower tear stain scores around the left and right eyes of sows in IMPROVED pens reflect lower levels of chronic stress in these animals (DeBoer *et al.* 2015; Telkänranta *et al.* 2016; Larsen *et al.* 2019). This is in agreement with the lower performance of stereotypical behaviour by sows in the IMPROVED pens described in this paper. Additionally, our companion paper (Lagoda *et al.* 2023), describes lower levels of haptoglobin (inflammatory marker), improved reproductive performance, and better health of piglets born to sows from IMPROVED pens. Moreover, these findings emphasise the potential of tear-stain scoring to discern cumulative benefits to welfare in a systems study. Our finding is similar to that of Telkänranta *et al.* (2016) where growing-finishing pigs tended to have lower tear-stain scores around the left but not the right eye in pens designed to improve welfare (e.g. equipped with manipulable objects made of fresh wood or polythene plastic), compared to control pens. Similarly, sows housed in pens with piglets and access to sisal ropes tended to have lower tear-staining scores than control sows with no access to rope (Telkänranta *et al.* 2016). Kinane *et al.* (2022) also recorded less tear staining around the left eye of sows housed in free lactation pens at weaning, compared to those confined to farrowing crates for the duration of lactation. Moreover, despite having access to manipulable objects (Astro 200 toy, chain/wood), sows in CONTROL pens still had higher tear-stain scores than sows in IMPROVED pens. Hence, it is possible that of all the enrichment items provided to sows in IMPROVED pens, straw had the most powerful impact on improving welfare and reducing stress levels, likely by improving gut fill and thereby satiety, and by enriching the overall diet, recently identified as an important factor for the welfare of pigs (Kobek-Kjeldager *et al.* 2022). Nonetheless, as this was a systems study, this effect cannot be ascribed to straw alone, as it is possible that other improvements in the pen also had partial beneficial effects that contributed to the lower scores for tear staining.

## Animal welfare implications and conclusion

The current study indicates that it is possible to improve sow welfare even within the constraints of fully slatted conventional gestation housing systems by implementing a number of modifications designed to lower chronic stress. It seems likely that the benefits accruing from the provision of enrichment resources and comfortable resting surfaces compensated for any potential detrimental effects of the increased aggression and associated stress caused by competition for straw provided in racks and rooting towers. Thus, implementing such improvements to pen design can be an effective alternative to major structural alterations, and has a cumulative beneficial effect in addressing both physical and psychological stressors experienced by sows.

## References

[r1] Arey DS and Edwards SA 1998 Factors influencing aggression between sows after mixing and the consequences for welfare and production. Livestock Production Science 56: 61–70. 10.1016/S0301-6226(98)00144-4

[r2] Bernardino T, Tatemoto P, de Moraes JE, Morrone B and Zanella AJ 2021 High fiber diet reduces stereotypic behavior of gilts but does not affect offspring performance. Applied Animal Behaviour Science 243: 105433.

[r3] Boyle LA, Regan D, Leonard FC, Lynch PB and Brophy P 2000 The effect of mats on the welfare of sows and piglets in the farrowing house. Animal Welfare 9: 39–48.

[r4] Calderon Diaz JA and Boyle LA 2014 Effect of rubber slat mats on the behaviour and welfare of group housed pregnant sows. Applied Animal Behaviour Science 151: 13–23. 10.1016/j.applanim.2013.11.016

[r5] Calderon Diaz JA, Fahey AG, KilBride AL, Green LE and Boyle LA 2013 Longitudinal study of the effect of rubber slat mats on locomotory ability, body, limb and claw lesions, and dirtiness of group housed sows. Journal of Animal Science 91: 3940–3954. 10.2527/jas.2012-5913 23881683

[r6] Calvert S, Haskell M, Wemelsfelder F, Lawrence AB and Mendl MT 1996 The effect of substrate-enriched and substrate-impoverished housing environments on the diversity of behaviour in pigs. Behaviour 133: 741–761.

[r7] Carroll GA, Boyle LA, Hanlon A, Palmer MA, Collins L, Griffin K, Armstrong D and O’Connell NE 2018 Identifying physiological measures of lifetime welfare status in pigs: Exploring the usefulness of haptoglobin, C- reactive protein and hair cortisol sampled at the time of slaughter. Irish Veterinary Journal 71. 10.1186/s13620-018-0118-0 PMC5833096 29507716

[r8] Casal-Plana N, Manteca, X, Dalmau A and Fàbrega E 2017 Influence of enrichment material and herbal compounds in the behaviour and performance of growing pigs. Applied Animal Behaviour Science 195: 38–43.

[r9] Cronin G and Wiepkema P 1984 An analysis of stereotyped behaviour in tethered sows. Annales de Recherches Vétérinaires 15: 263–270. 6541447

[r10] DeBoer S, Garner J, McCain R, Lay Jr D, Eicher S and Marchant-Forde J 2015 An initial investigation into the effects of isolation and enrichment on the welfare of laboratory pigs housed in the PigTurn® system, assessed using tear staining, behaviour, physiology and haematology. Animal Welfare 24: 15–27.

[r11] DeBoer S and Marchant-Forde J 2013 Tear staining as a potential welfare indicator in pigs. Proceedings of the 47th Congress of the International Society for Applied Ethology. 2-6 June, 2013, Florianopolis, Brazil.

[r12] Edwards LE, Plush KJ, Ralph CR, Morrison RS, Acharya RY and Doyle RE 2019 Enrichment with lucerne hay improves sow maternal behaviour and improves piglet survival. Animals 9: 558. 31443165 10.3390/ani9080558 PMC6719939

[r13] Elmore MR, Garner JP, Johnson AK, Richert BT and Pajor EA 2010 A flooring comparison: The impact of rubber mats on the health, behavior, and welfare of group-housed sows at breeding. Applied Animal Behaviour Science 123: 7–15.

[r14] Elmore MRP, Garner JP, Johnson AK, Kirkden RD, Richert BT and Pajor EA 2011 Getting around social status: Motivation and enrichment use of dominant and subordinate sows in a group setting. Applied Animal Behaviour Science 133: 154–163.

[r15] Herman J, McKlveen J, Ghosal S, Kopp B, Wulsin A, Makinson R, Scheimann J and Myers B 2016 Regulation of the hypothalamic‐pituitary‐adrenocortical stress response. Comprehensive Physiology 6: 603–621. 27065163 10.1002/cphy.c150015 PMC4867107

[r16] Horback KM, Pierdon MK and Parsons TD 2016 Behavioral preference for different enrichment objects in a commercial sow herd. Applied Animal Behaviour Science 184: 7–15.

[r17] Jensen P 1988 Diurnal rhythm of bar-biting in relation to other behavior in pregnant sows. Applied Animal Behaviour Science 21: 337–346. 10.1016/0168-1591(88)90068-8

[r18] Kinane O, Butler F and O’Driscoll K 2022 Freedom to move: Free lactation pens improve sow welfare. Animals 12: 1762. 35883309 10.3390/ani12141762 PMC9311877

[r19] Kobek-Kjeldager C, Schönherz AA, Canibe N and Pedersen LJ 2022 Diet and microbiota-gut-brain axis in relation to tail biting in pigs: A review. Applied Animal Behaviour Science 246: 105514.

[r20] Lagoda M, O’Driscoll K, Marchewka J, Foister S, Turner S and Boyle L 2021a Associations between skin lesion counts, hair cortisol concentrations and reproductive performance in group housed sows. Livestock Science 246: 104463.

[r21] Lagoda ME, Boyle LA, Marchewka J and O’Driscoll K 2021b Early detection of locomotion disorders in gilts using a novel visual analogue scale; associations with chronic stress and reproduction. Animals 11: 2900. 34679922 10.3390/ani11102900 PMC8532660

[r22] Lagoda ME, Marchewka J, O’Driscoll K and Boyle LA 2022 Risk factors for chronic stress in sows housed in groups, and associated risks of prenatal stress in their offspring. Frontiers in Veterinary Science 9. 10.3389/fvets.2022.883154 PMC9039259 35498729

[r23] Lagoda ME, O’Driscoll K, Galli MC, Ceron JJ, Ortin-Bustillo A, Marchewka J and Boyle LA 2023 Indicators of improved gestation housing of sows. Part II: Effects on physiological measures, reproductive performance and health of the offspring. Animal Welfare 32, 10.1017/awf.2023.48 PMC10936399 38487422

[r24] Larsen M, Gustafsson A, Marchant-Forde J and Valros A 2019 Tear staining in finisher pigs and its relation to age, growth, sex and potential pen level stressors. Animal 13: 1704–1711. 30614436 10.1017/S1751731118003646

[r25] Lawrence AB, McLean KA, Jarvis S, Gilbert CL and Petherick JC 1997 Stress and parturition in the pig. Reproduction in Domestic Animals 32: 231–236. 10.1111/j.1439-0531.1997.tb01287.x

[r26] Marchant J and Broom D 1996 Effects of dry sow housing conditions on muscle weight and bone strength. Animal Science 62: 105–113.

[r27] Martinez-Miro S, Tecles F, Ramon M, Escribano D, Hernandez F, Madrid J, Orengo J, Martinez-Subiela S, Manteca X and Ceron JJ 2016 Causes, consequences and biomarkers of stress in swine: an update. BMC Veterinary Research 12. 10.1186/s12917-016-0791-8 PMC4992232 27543093

[r28] Merlot E, Calvar C and Prunier A 2017 Influence of the housing environment during sow gestation on maternal health, and offspring immunity and survival. Animal Production Science 57: 10.1071/An15480

[r29] Mkwanazi MV, Ncobela CN, Kanengoni AT and Chimonyo M 2019 Effects of environmental enrichment on behaviour, physiology and performance of pigs—A review. Asian-Australasian Journal of Animal Sciences 32: 1. 28728387 10.5713/ajas.17.0138 PMC6325398

[r30] Oczak M, Maschat K, Berckmans D, Vranken E and Baumgartner J 2015 Classification of nest-building behaviour in non-crated farrowing sows on the basis of accelerometer data. Biosystems Engineering 140: 48–58. 10.1016/j.biosystemseng.2015.09.007

[r31] Olsson A-C, Samuelsson O, Collins E and Boom C 1993 Grouping studies of lactating and newly weaned sows. Livestock Environment 4: 475–482.

[r32] Ostovic M, Rafaj RB, Mencik S, Kabalin AE, Grahovac J, Matkovic K, Vucinic M, Nenadovic K, Zaja IZ and Pavicic Z 2017 The effect of rubber slat mats on cortisol concentrations in stall-housed gilts. Veterinarski Arhiv 87: 185–196.

[r33] Perrin W and Bowland J 1977 Effects of enforced exercise on the incidence of leg weakness in growing boars. Canadian Journal of Animal Science 57: 245–253.

[r34] Philipot J, Pluvinage P, Cimarosti I, Sulpice P and Bugnard F 1994 Risk factors of dairy cow lameness associated with housing conditions. Veterinary Research 25: 244–248. 8038793

[r35] Quesnel H, Peuteman B, Pere MC, Louveau I, Lefaucheur L, Perruchot MH, Prunier A, Meunier-Salaun MC, Gardan-Salmon D, Gondret F and Merlot E 2019 Effect of environmental enrichment with wood materials and straw pellets on the metabolic status of sows during gestation. Livestock Science 229: 43–48. 10.1016/j.livsci.2019.09.005

[r36] Roy C, Lippens L, Kyeiwaa V, Seddon YM, Connor LM and Brown JA 2019 Effects of enrichment type, presentation and social status on enrichment use and behaviour of sows with electronic sow feeding. Animals 9: 369. 31216708 10.3390/ani9060369 PMC6616966

[r37] Salak-Johnson JL 2017 Social status and housing factors affect reproductive performance of pregnant sows in groups. Molecular Reproduction and Development 84: 905–913. 10.1002/mrd.22846 28763574

[r38] Schenck E, McMunn K, Rosenstein D, Stroshine R, Nielsen B, Richert B, Marchant-Forde J and Lay Jr D 2008 Exercising stall-housed gestating gilts: Effects on lameness, the musculo-skeletal system, production, and behavior. Journal of Animal Science 86: 3166–3180. 18567722 10.2527/jas.2008-1046

[r39] Spoolder HAM, Geudeke MJ, Van der Peet-Schwering CMC and Soede NM 2009 Group housing of sows in early pregnancy: a review of success and risk factors. Livestock Science 125: 1–14. 10.1016/j.livsci.2009.03.009

[r40] Stewart CL, O’Connell NE and Boyle L 2008 Influence of access to straw provided in racks on the welfare of sows in large dynamic groups. Applied Animal Behaviour Science 112: 235–247. 10.1016/j.applanim.2007.09.006

[r41] Studnitz M, Jensen MB and Pedersen LJ 2007 Why do pigs root and in what will they root?: A review on the exploratory behaviour of pigs in relation to environmental enrichment. Applied Animal Behaviour Science 107: 183–197.

[r42] Tatemoto P, Bernardino T, Rodrigues FAML and Zanella AJ 2019 Does high stereotypic behavior expression affect productivity measures in sows? Revista Brasileira de Zootecnia 48: e20180135.

[r43] Telkänranta H, Marchant-Forde JN and Valros A 2016 Tear staining in pigs: a potential tool for welfare assessment on commercial farms. Animal 10: 318–325. 26303891 10.1017/S175173111500172X

[r44] Turner SP, Farnworth MJ, White IMS, Brotherstone S, Mendl M, Knap P, Penny P and Lawrence AB 2006 The accumulation of skin lesions and their use as a predictor of individual aggressiveness in pigs. Applied Animal Behaviour Science 96: 245–259. 10.1016/j.applanim.2005.06.009

[r45] Tuyttens FAM 2005 The importance of straw for pig and cattle welfare: a review. Applied Animal Behaviour Science 92: 261–282.

[r46] Tuyttens FAM, Wouters F, Struelens E, Sonck B and Duchateau L 2008 Synthetic lying mats may improve lying comfort of gestating sows. Applied Animal Behaviour Science 114: 76–85. 10.1016/j.applanim.2008.01.015

[r47] van de Weerd H and Ison S 2019 Providing effective environmental enrichment to pigs: How far have we come? Animals 9: 254. 31117191 10.3390/ani9050254 PMC6562439

[r48] Whittaker X, Edwards S, Spoolder H, Lawrence A and Corning S 1999 Effects of straw bedding and high fibre diets on the behaviour of floor fed group-housed sows. Applied Animal Behaviour Science 63: 25–39.

[r49] Whittaker X, Spoolder H, Edwards S, Lawrence A and Corning S 1998 The influence of dietary fibre and the provision of straw on the development of stereotypic behaviour in food restricted pregnant sows. Applied Animal Behaviour Science 61: 89–102.

[r50] Winkel C, von Meyer-Höfer M and Heise H 2020 Understanding German pig farmers’ intentions to design and construct pig housing for the improvement of animal welfare. Animals 10: 1760. 32998317 10.3390/ani10101760 PMC7600590

